# Burn Extent and Fitzpatrick Skin Tone Assessment from Clinical Photographs: Systematic and Random Error in Multimodal Large Language Models

**DOI:** 10.3390/bioengineering13091000

**Published:** 2026-08-28

**Authors:** Ibrahim Güler, Armin Kraus, Gerrit Grieb, Henrik Stelling

**Affiliations:** 1 Department of Plastic, Aesthetic and Hand Surgery, Otto-von-Guericke University, 39120 Magdeburg, Germany; armin.kraus@med.ovgu.de; 2 Department of Health Management, Friedrich-Alexander-Universität Erlangen-Nürnberg, 90403 Nuremberg, Germany; henrik.stelling@fau.de; 3 Department of Plastic Surgery and Hand Surgery, Gemeinschaftskrankenhaus Havelhöhe, 14089 Berlin, Germany; gerritgrieb@gmx.de; 4 Department of Plastic Surgery and Hand Surgery, Medical Faculty, Rheinisch-Westfälische Technische Hochschule Aachen, 52074 Aachen, Germany; 5 Practices for Nuclear Medicine, 12157 Berlin, Germany

**Keywords:** burn assessment, total body surface area, clinical photography, multimodal large language model, artificial intelligence, medical image analysis, measurement error, reproducibility, Fitzpatrick skin tone, health equity

## Abstract

Background: Burn extent guides triage, transfer and fluid resuscitation, yet its clinical estimation is imprecise and observer-dependent. Multimodal large language models (MLLMs) process clinical photographs without task-specific training, but their error has rarely been separated into systematic and random components or their performance across skin tones characterized. Methods: Three state-of-the-art MLLMs (Gemini 3.1 Pro, GPT-5.6 Sol, and Fable 5) each assessed 153 burn photographs five times under an identical prompt. The tasks were as follows: burned proportion of the imaged field, against an expert-guided pixel-wise segmentation (tolerance ± 10 percentage points, pp); burned percentage of total body surface area (TBSA), against physician consensus (±2 pp); and binary Fitzpatrick skin tone (FST; light I–III versus dark IV–VI). The first of these was the primary endpoint. Results: The primary endpoint was in the range of 32.5–70.2%, with TBSA at 69.5–77.5%. All models compressed the estimation range (slopes 0.58–0.76, intercepts +10.0 to +22.2 pp); one multiplicative constant per model brought errors differing more than twofold into a 1.4 pp range. Across repeated queries, the median within-image range was 5.0–25.0 pp; averaging the five answers reduced error by only 0.24–2.22 pp. FST accuracy was 83.8–91.9% against a majority-class baseline of 81.0%. Conclusions: Averaging repeated answers removes only the smaller, random component; the larger, systematic one persists and requires calibration against reference data before clinical use can be considered.

## 1. Introduction

The extent of a burn injury, expressed as the percentage of total body surface area (TBSA) affected, is together with burn depth one of the two quantitative variables that guide early management. It informs the transfer decision, the calculation of resuscitation volume, the prognostic assessment on admission and other early choices. Despite this central role, estimation of burn size at first contact is not reliable. Inter-rater reliability of chart-based estimation is limited even under standardized conditions, and agreement between the TBSA determined at referring units and at receiving burn centers is consistently low, with overestimation predominating in most reported series [1,2].

Overestimation has clinical consequences: the resuscitation volume is calculated directly from the estimated TBSA, so an error in the estimate carries through into the prescribed fluid, and overestimation has been associated with volumes substantially in excess of what a correct estimate would have indicated [3,4]. Even between emergency physicians and burn-unit staff assessing the same patients, TBSA estimates frequently differ, with overestimation being more common than underestimation [5].

The methods in routine use, Wallace’s rule of nines, the rule of palms and the Lund–Browder chart, are quick but coarse, and estimation errors are common at all levels of experience [6,7,8]. Objective alternatives exist, including pixel-by-pixel planimetry on admission photographs, which has been shown to yield significantly lower TBSA estimates than either referring emergency physicians or consultant plastic surgeons; three-dimensional surface capture, which accounts for body surface curvature and therefore measures wound area more accurately than two-dimensional projection; and, for depth, laser Doppler imaging [9,10,11]. Cost, availability and workflow constraints have nevertheless limited their adoption outside specialized centers. Automated image analysis has therefore long been pursued as a way to remove observer judgment from the estimate, and supervised deep learning has dominated the field in recent years. Convolutional neural networks (CNNs) trained for the task can delineate the burned region from color photographs and derive the burned percentage of TBSA from it and can classify burn depth [12,13,14]. Where such a model has been compared with current methods of burn assessment, the results were encouraging [15]. A systematic review of this literature nevertheless identified recurring limitations: small and homogeneous datasets, limited external validation, and in particular restricted diversity of skin tone, although more recent studies have begun to report stratified performance across Fitzpatrick types [16,17].

Multimodal large language models (MLLMs) differ from this paradigm in a practically relevant way. They are general-purpose AI (GPAI) systems, not models trained for a single task: they process an image together with a free-text instruction and return a natural-language answer, without task-specific training and without local infrastructure. These models are already accessible to clinicians and patients without institutional deployment, so their use does not wait for validation. How they perform is already a practical question [18,19]. Two studies have applied general-purpose MLLMs of the GPT family to clinical photographs of burns. In a prospective emergency-department study, region-level TBSA estimates were non-inferior to those of emergency physicians against an expert-panel reference, whereas burn-depth classification was substantially worse and systematically underestimated deeper burns [20]. A second study combined photographs with ultrasound tissue Doppler imaging and expert-written prompts to classify burn depth within an electronic medical record [21]. It addressed burn depth rather than burn extent and reduced skin tone as a potential confounder by cropping photographs to the wound area, a preprocessing step the authors acknowledge may not always be feasible in routine clinical practice.

Three properties are insufficiently characterized. The first is calibration. Performance on an estimation task can be judged along two axes: how close each individual estimate lies to the true value and how well the estimates preserve the ordering of cases. The two can diverge, and rank correlations reward the second even where the first fails. Clinically, only the first is usable: a burn is managed on the percentage assigned to that patient, from resuscitation volume to transfer and prognosis, and not on where that patient sits relative to others. The second is reproducibility. MLLM outputs are stochastic, and repeated identical queries can yield different answers even when the prompt, model and parameters are held constant [22]. Many published evaluations report performance from a single query per case (single-run evaluation), which cannot distinguish what a model typically answers from what it happened to answer once. That requires repeated inference on identical inputs (multi-run evaluation). Promising benchmark performance does not necessarily translate into clinical benefit [23,24,25]. The third property is equity across Fitzpatrick skin tone (FST) [26]. Performance disparities across skin tones have been reported for image-based artificial intelligence, with models generally performing less well on darker skin [27]. Since burn appearance is itself influenced by baseline pigmentation, it remains unclear whether similar disparities extend to burn assessment.

Building on these evaluations, the present study takes a point-in-time assessment of where three state-of-the-art (SOTA) MLLMs stand in burn assessment on 153 burn photographs under a multi-run design. It asks how large the error is, whether that error has a structure, and how reliably these models answer at all, and from that describes the current position and what would have to change before such systems could be considered for use. It thereby complements earlier work on general-purpose models in burn assessment, including the evaluations discussed above, which proceeded under a different design [20,21].

## 2. Materials and Methods

This section sets out the study design, the dataset and the three reference standards, the models and the inference protocol, and the outcome measures and statistical analysis.

### 2.1. Study Design

This cross-sectional benchmarking study evaluated three SOTA MLLMs on a fixed, retrospectively assembled set of burn photographs, with five independent inference runs per model and image. Three tasks were assessed per image: the proportion of the imaged field occupied by burn, the burned percentage of TBSA, and the binary FST. The primary endpoint was the proportion of burned-image-area estimates falling within ±10 percentage points (pp) of the reference. All other analyses were secondary. Both tolerances were set a priori and clinically rather than statistically. For TBSA, the ±2 pp tolerance is small relative to the disagreement documented between referring institutions and burn centers [1,3] and to the TBSA steps that structure referral and resuscitation decisions. For burned image area, no clinical convention exists, and ±10 pp was set as a pragmatic bound proportionate to a quantity that is framed by the photograph rather than by whole-body anatomy and therefore takes much larger values than TBSA. Within-tolerance rates at alternative tolerances are reported as a sensitivity check. Reporting was informed by the Checklist for Artificial Intelligence in Medical Imaging (CLAIM) [28] and the Standards for Reporting Diagnostic Accuracy studies using Artificial Intelligence (STARD-AI) [29], although neither applies in full to the present design, since both address task-specific model development or patient-level diagnostic accuracy evaluation rather than benchmarks of GPAI systems on a fixed public image set.

### 2.2. Dataset

The dataset (Extended Burn Image Segmentation; EBIS) is available in a public online repository [30] associated with prior work [31,32,33,34,35]. It provides color photographs of burn injuries together with pixel-wise binary annotations of the burned region, that is, a binary semantic segmentation in which every pixel is assigned to one of two classes, burn or non-burn. The annotations were produced manually in consultation with a clinical burn expert. The analysis set comprised 153 images, covering different body regions including the head, the trunk, and the upper and lower extremities. The images were acquired under heterogeneous conditions, with varying illumination, different source cameras, and varying camera-to-subject distances [30,34].

### 2.3. Reference Standards

For burned image area (Task 1), the reference was the burned fraction of the image, obtained by pixel counting on the binary annotation at native resolution. The annotation contains two dominant pixel classes, so any threshold between them yields the same result. Antialiased edge pixels account for 0.03% of all pixels. Because the reference is obtained by counting rather than by estimation, Task 1 is reported as accuracy.

For TBSA (Task 2), two physicians assessed all images in a single joint reading and agreed one consensus value per case, resolving discrepancies by discussion. TBSA was defined as the percentage of the whole-body surface area accounted for by the burn visible in the photograph. For brevity, “TBSA” denotes this burned percentage throughout, and deviations from it are given in percentage points. The raters worked under the same instruction given to the models. Because this reference is itself an expert estimate, Task 2 is reported as agreement rather than accuracy. Burned image area and TBSA differ in their denominator and are kept terminologically separate throughout.

For Fitzpatrick skin tone (FST, Task 3), the two physicians assessed the perilesional unburned skin in the same joint reading and assigned it to one of two categories, light or dark, corresponding to Fitzpatrick types I–III and IV–VI. Accuracy is used for this task in its conventional sense as the proportion of correct classifications. A note on terminology is needed here, because skin type, phototype and skin tone are not used consistently in this literature. The Fitzpatrick classification was defined by the response of the skin to ultraviolet exposure, but the label is also widely applied as a visual descriptor of pigmentation, particularly in image-based work, where the sun-exposure history on which it was built is not available and where it correlates only weakly with objective measurements of skin color. The present assignment was made from the photograph alone, with no information on sun sensitivity, sunburn or tanning response, and is therefore a visual reading. The term Fitzpatrick skin tone (FST) is used throughout to name precisely that: the visually assessed tone of the unburned skin, grouped by the Fitzpatrick dichotomy, rather than a phototype in the sense of the original classification [17,26,27,36,37].

### 2.4. Models and Inference Protocol

Three SOTA MLLMs were evaluated: Gemini 3.1 Pro (Google DeepMind, Mountain View, CA, USA), GPT-5.6 Sol (OpenAI, San Francisco, CA, USA) and Claude Fable 5 (Anthropic, San Francisco, CA, USA). All models were accessed through their respective first-party web interfaces in July 2026 using default inference configurations. No application programming interface (API) or other programmatic access was used, and the web interfaces expose no temperature or related sampling controls. This setup reflects the deployment context in which general-purpose MLLMs are predominantly accessed. No model was fine-tuned, system-prompted or primed with in-context examples.

Each model was queried five times per image, with each run an independent request in a new context, giving 6885 model outputs in total (153 images × 3 tasks × 3 models × 5 runs), none missing or invalid. The photograph was the sole visual input. The binary annotations were never shown to the models and served exclusively to derive the Task 1 reference. An identical prompt was used for every query and asked for three items per image (File S1). For burned image area, the models reported the percentage of the photograph covered by burn, counting the burned region only, the same denominator as the reference. For TBSA, they reported the percentage of the whole-body surface area accounted for by the visible burn, under the explicit instruction to assume no burn outside the imaged field. For FST, they returned a binary classification using the same light and dark dichotomy as the reference.

### 2.5. Outcome Measures

For both continuous tasks (Task 1, burned image area, and Task 2, TBSA), the metrics were the mean absolute error (MAE, in pp), the mean signed error as the measure of directional bias, the within-tolerance rate, the 2.5th–97.5th percentile band of the 765 single-query signed errors of each model (153 images × 5 runs), Spearman’s rank correlation between estimate and reference (Task 1 only, where the reference is not itself an estimate), the intraclass correlation coefficient ICC(A,1) across the five runs, the within-image coefficient of variation (CV), and the within-image range across the five runs [38,39]. The tolerance was ±10 pp for Task 1 and ±2 pp for Task 2, as specified in Section 2.1. Bland–Altman analysis was performed for Task 2 only. The deviation on Task 1 is quantified by regression. For the Bland–Altman analysis, the limits of agreement are the 2.5th and 97.5th percentiles of the differences rather than mean ±1.96 standard deviations (SDs), and the bias is the median difference [40].

For the binary task (Task 3, FST) the metrics were accuracy, sensitivity (recall), specificity, positive predictive value (PPV; precision), negative predictive value (NPV), the F1 score, and, because the classes are imbalanced, balanced accuracy and the Matthews correlation coefficient (MCC), together with Fleiss’ κ across the five runs and the majority-vote accuracy, which is the accuracy of the per-image majority classification [41]. The dark skin tone was defined as the positive class. Because accuracy on an imbalanced task can be attained without any discrimination, each model was also compared with a majority-class baseline, with the accuracy obtained by always answering “light”.

MAE, the mean signed error, the within-tolerance rate and the Task 3 diagnostic metrics were computed within each of the five runs and the five values then averaged, so each estimates expected single-query performance. The ICC, the CV, Fleiss’ κ, the majority-vote accuracy, the error-spread percentiles and the within-image range are by definition quantities across runs and are not run-averaged. The proportion of squared error removed by averaging is one minus the ratio of two quantities, both taken over the 153 images: the mean squared error of the mean of the five runs divided by the mean squared error of a single run averaged over the five runs. The rank correlation, the regression, the calibration constant and the Bland–Altman analysis use the mean of the five runs per image. The Task 3 inferential comparisons use the per-image majority vote.

### 2.6. Statistical Analysis

Confidence intervals (CIs) are case-level non-parametric bootstrap intervals: the 153 images were resampled with replacement 2000 times with a fixed seed and the run-averaged metric recomputed in each resample, which preserves the dependence among the five runs of one image. Model comparisons are paired, all models having seen the same images: for the continuous tasks, an omnibus Friedman test on the per-image absolute error with post hoc Wilcoxon signed-rank tests; for Task 3, an omnibus Cochran’s Q on the per-image majority-vote correctness with post hoc exact McNemar tests. Each model was additionally compared with the majority-class baseline by exact McNemar on the same 153 images. Subgroup comparisons by FST are unpaired, because the two subgroups are different images, and use the Mann–Whitney U test on the per-image absolute error, with bootstrap CIs for the difference of the median per-image errors, and Fisher’s exact test for Task 3. Subgroups were defined by the consensus physician reference and never by a model estimate. Risk differences are reported for binary outcomes. Spearman’s rank correlation was additionally used to test whether the within-image SD across runs scaled with the magnitude of the estimate. The Benjamini–Hochberg procedure was applied within each family of three post hoc tests, omnibus tests were not corrected, and both *p* and *q* are reported for every comparison with an effect size appropriate to the comparison: the median per-image paired difference in absolute error, the difference of median per-image errors, or the risk difference [42]. The significance level was two-sided α = 0.05.

Two analyses are post hoc. The first is the estimation-scale analysis of Task 1, comprising ordinary least-squares regression of the run-averaged estimate on the reference with the slope tested against 1, together with a calibration constant k defined as the median ratio of reference to the estimate, after which the error is recomputed. Because k is fitted and evaluated on the same images, the post-rescaling error is optimistic. A leave-one-out cross-validation (LOOCV) is reported below. The second is an adjustment of the FST subgroup comparison for burn magnitude: an ordinary least-squares model of the per-image absolute error, or of majority-vote correctness, on skin tone with both reference values as covariates, with case-level bootstrap intervals for the adjusted difference. For the classification task this is a linear probability model, and the estimate is an adjusted risk difference. It is reported as an estimate rather than as an additional test, so the family of corrected comparisons is unchanged.

All analyses were performed in Python 3.12 (Python Software Foundation, Wilmington, DE, USA) with standard statistical and data visualization libraries.

## 3. Results

The results follow the order of the analysis: the reference standard distributions and task-level performance first, then each of the three tasks in turn, then the systematic and the random component of the error and the structure of the TBSA agreement, and finally the skin tone subgroups and the comparisons between models.

### 3.1. Reference Standard Distributions

The reference burned image area had a median of 17.75% (interquartile range, IQR, 9.59–28.84), a mean of 22.37% (SD 16.74) and a range of 1.59–82.56%. The reference TBSA had a median of 2.50% (IQR 0.60–6.00), a mean of 4.49% (SD 4.89) and a range of 0.10–19.00%. The reference FST was light (I–III) in 124 of 153 images (81.0%) and dark (IV–VI) in 29 (19.0%).

### 3.2. Task-Level Performance

**Figure 1:**
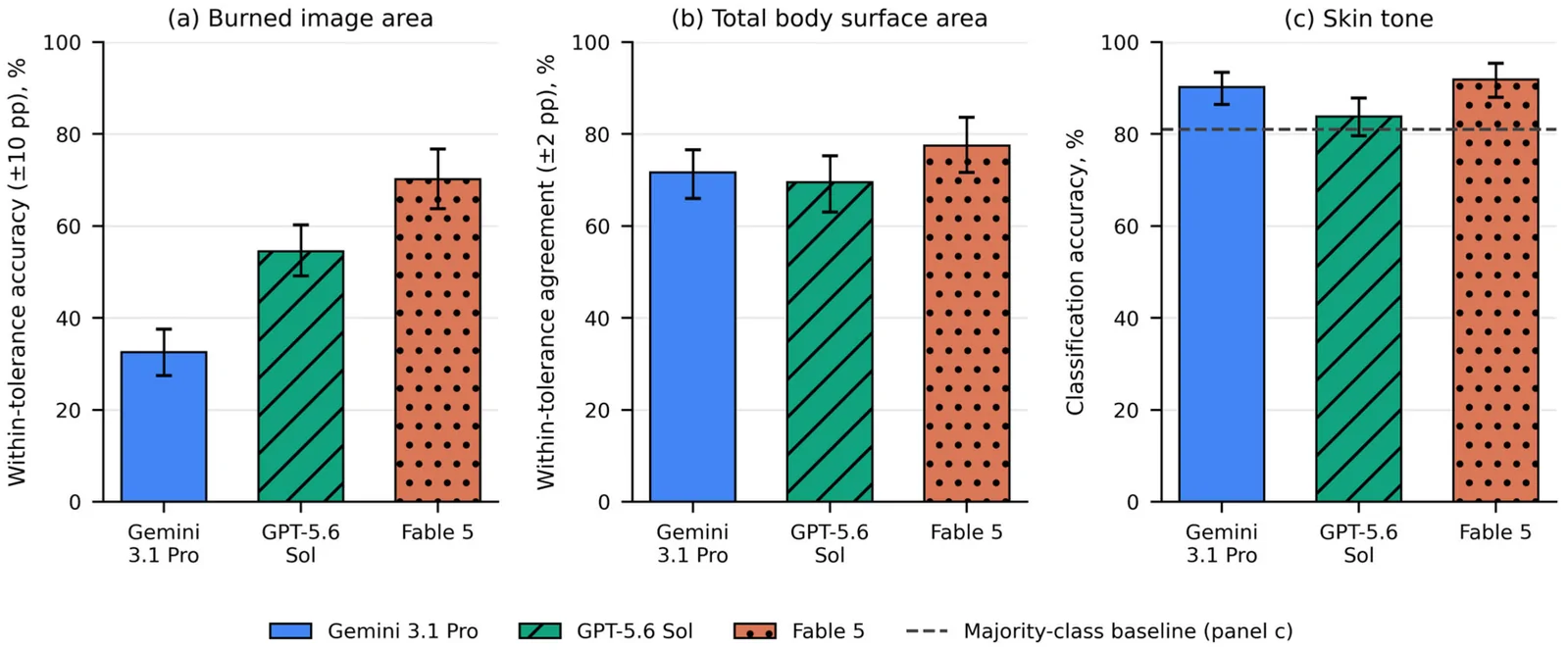
Task-level performance of the three models. (**a**) Proportion of burned-image-area estimates within ±10 percentage points of the pixel-wise reference standard; this is the primary endpoint. (**b**) Proportion of TBSA estimates within ±2 percentage points of the consensus physician reference. (**c**) Accuracy of binary FST classification; the dashed line marks the majority-class baseline of 81.0%, the accuracy attainable by always predicting the light skin tone. Bars show the metric computed within each of the five runs and then averaged; error bars are 95% case-level bootstrap confidence intervals. pp = percentage points; TBSA = total body surface area; FST = Fitzpatrick skin tone.

Figure 1 summarizes the three tasks as the proportion of answers that were within tolerance or, for FST, correct. On the primary endpoint, the share of burned-image-area estimates within ±10 pp of the pixel-wise reference, performance ranged from 32.5% (95% CI 27.8 to 37.6) for Gemini 3.1 Pro through 54.5% (49.4 to 59.9) for GPT-5.6 Sol to 70.2% (63.8 to 76.1) for Fable 5 (Table 1, Panel A). On TBSA the three models were closer together and higher, at 71.6% (66.4 to 76.6), 69.5% (63.3 to 75.4) and 77.5% (71.1 to 83.1) within ±2 pp (Table 1, Panel B). FST classification accuracy was 90.2% (86.4 to 93.6), 83.8% (79.6 to 87.8) and 91.9% (88.1 to 95.3), against a majority-class baseline of 81.0% (Table 2). No conclusion depends on the chosen tolerance. On burned image area the ordering of the three models was unchanged at every tolerance examined, with within-tolerance rates at ±5, ±15 and ±20 pp of 15.4%, 48.6% and 60.8% for Gemini 3.1 Pro, 32.7%, 68.5% and 79.1% for GPT-5.6 Sol and 46.5%, 86.0% and 93.5% for Fable 5. On TBSA the corresponding rates at ±1 and ±3 pp were 54.9% and 80.5%, 53.9% and 79.9%, and 63.0% and 85.5%. Fable 5 ranked first at both, and the two remaining models lay within 1.1 pp of each other, consistent with the non-significant difference between them on this task.

### 3.3. Burned Image Area

MAE differed substantially between models: 18.76 pp (17.10 to 20.45) for Gemini 3.1 Pro, 12.30 pp (11.06 to 13.57) for GPT-5.6 Sol and 7.91 pp (6.84 to 9.13) for Fable 5 (Table 1, Panel A). All three overestimated on average, with mean signed errors of +16.80 pp (14.84 to 18.86) for Gemini 3.1 Pro, +4.49 pp (2.52 to 6.44) for GPT-5.6 Sol and +2.20 pp (0.60 to 3.77) for Fable 5. Overestimation was near-universal for Gemini 3.1 Pro, whose mean of the five runs exceeded the reference in 148 of 153 images (96.7%) and was less pronounced for GPT-5.6 Sol (105 of 153, 68.6%) and Fable 5 (97 of 153, 63.4%).

The dispersion of individual answers was wide on this scale. The central 95% of the 765 single-query signed errors spanned −12.5 to +54.2 pp for Gemini 3.1 Pro, −33.8 to +37.8 pp for GPT-5.6 Sol and −20.5 to +23.2 pp for Fable 5. The narrowest of these bands was thus 43.7 pp wide and the widest 71.6 pp.

### 3.4. Total Body Surface Area

Agreement with the physician reference was closer in absolute terms and more homogeneous between models (Table 1, Panel B). MAE was 2.00 pp (1.70 to 2.32) for Gemini 3.1 Pro, 1.82 pp (1.54 to 2.13) for GPT-5.6 Sol and 1.55 pp (1.29 to 1.84) for Fable 5. Directional bias differed in sign: Gemini 3.1 Pro returned values above the physician reference on average (+0.29 pp, −0.09 to 0.68), while Fable 5 (−0.72 pp, −1.07 to −0.39) and GPT-5.6 Sol (−1.11 pp, −1.47 to −0.79) returned values below it. The proportion of images on which the mean of the five runs exceeded the reference was 91 of 153 (59.5%) for Gemini 3.1 Pro, 34 of 153 (22.2%) for GPT-5.6 Sol and 67 of 153 (43.8%) for Fable 5. The central 95% of single-query errors spanned −7.0 to +10.0 pp for Gemini 3.1 Pro, −7.0 to +4.0 pp for GPT-5.6 Sol and −7.0 to +3.0 pp for Fable 5.

### 3.5. FST Classification

Sensitivity for the dark skin tone was consistently lower than specificity for the light skin tone (Table 2): 73.8% (63.0 to 84.2) for Gemini 3.1 Pro, 82.1% (70.7 to 92.0) for GPT-5.6 Sol and 84.1% (71.6 to 94.8) for Fable 5, against specificities of 94.0% (90.4 to 97.1), 84.2% (79.5 to 88.7) and 93.7% (89.8 to 97.0). PPV was correspondingly limited, from 55.7% to 76.5%, while NPV was high throughout, from 93.9% to 96.2%, reflecting the 19.0% prevalence of the dark skin tone. F1 scores were 73.7%, 66.0% and 79.9%. GPT-5.6 Sol tended to over-call the dark skin tone relative to its true frequency of 29 of 153, returning between 34 and 51 dark classifications per run, whereas Gemini 3.1 Pro returned 26 to 35 and Fable 5 27 to 35. Majority-vote accuracies were 90.8% for Gemini 3.1 Pro, 85.6% for GPT-5.6 Sol and 92.8% for Fable 5. Run-averaged balanced accuracy was 83.9% for Gemini 3.1 Pro, 83.1% for GPT-5.6 Sol and 88.9% for Fable 5, against 50.0% for the majority-class baseline, and run-averaged MCC was 0.68, 0.58 and 0.75. MCC is undefined for the baseline, which returns a single class.

### 3.6. Systematic Error: Estimation Scale and Its Correctability

**Figure 2:**
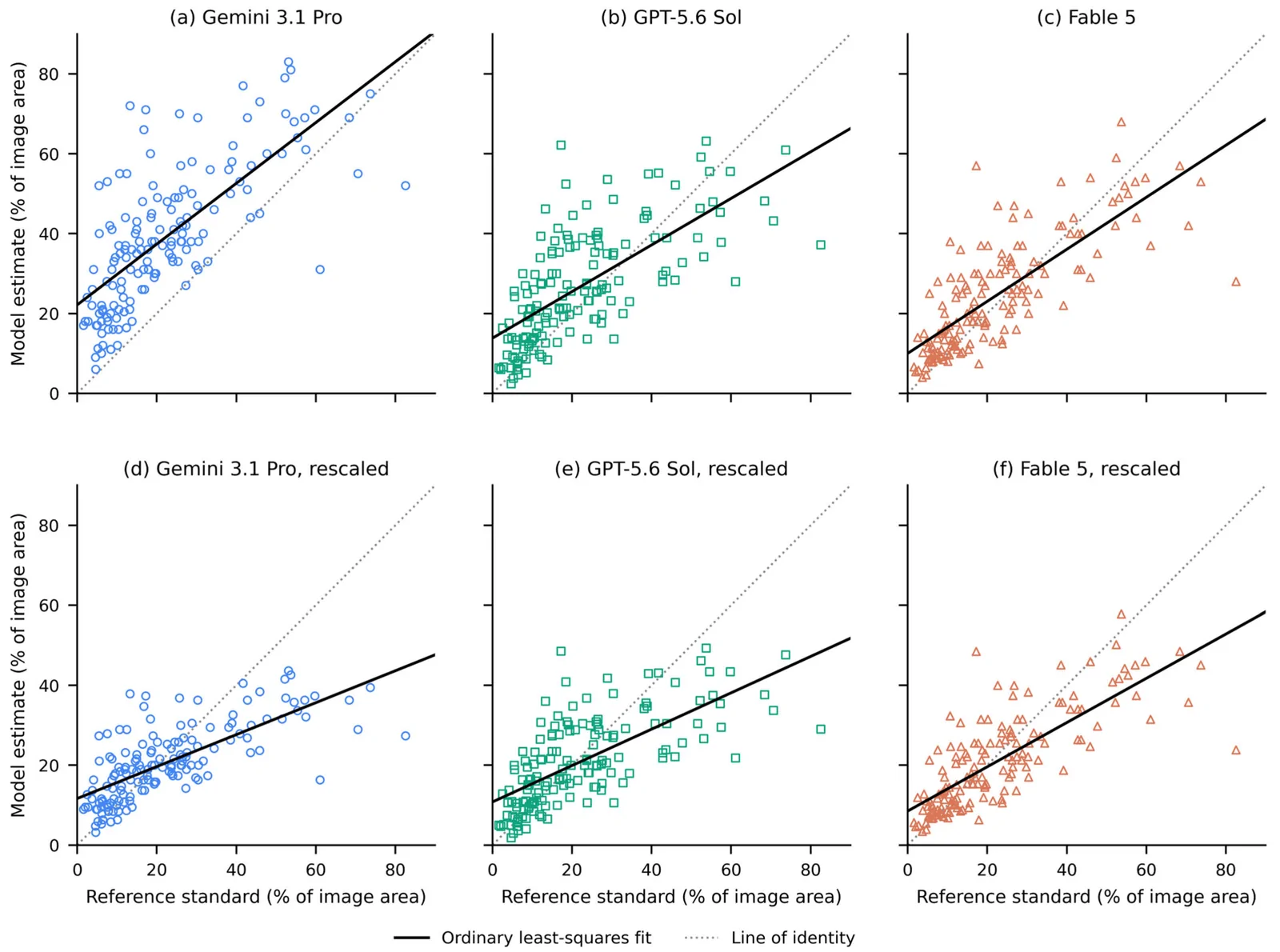
Estimation scale for burned image area. (**a**–**c**) Model estimate, the mean of the five runs, plotted against the pixel-wise reference standard for Gemini 3.1 Pro, GPT-5.6 Sol and Fable 5. The dotted line is the line of identity; the solid line is the ordinary least-squares fit. Slope, intercept and coefficient of determination were 0.76, +22.2 pp and 0.52 in (**a**); 0.58, +13.8 pp and 0.47 in (**b**); and 0.65, +10.0 pp and 0.60 in (**c**), with all three slopes differing from unity at *p* < 0.001. A slope below unity with a positive intercept indicates compression of the estimation range. (**d**–**f**) The same estimates after multiplication by the model-specific calibration constant k, the median ratio of reference to estimate, on identical axes. Panels (**d**–**f**) follow the same model order. The constants were 0.53, 0.78 and 0.85, and the mean absolute error of the averaged estimate fell from 17.82 to 8.34 pp in (**d**), from 10.08 to 8.72 pp in (**e**) and from 7.67 to 7.34 pp in (**f**).

Regression of the run-averaged estimate on the pixel-wise reference showed the same pattern in all three models (Figure 2a–c): slopes below unity, at 0.76 (0.62 to 0.93) for Gemini 3.1 Pro, 0.58 (0.48 to 0.70) for GPT-5.6 Sol and 0.65 (0.53 to 0.77) for Fable 5, all differing from 1 at *p* < 0.001, combined with positive intercepts of +22.2 pp (+18.3 to +25.6), +13.8 pp (+11.1 to +16.4) and +10.0 pp (+7.5 to +12.6), with values of R^2^ of 0.52, 0.47 and 0.60. Small burns were therefore overestimated and large burns underestimated by every model. Rank correlation between the run-averaged estimate and the reference was nevertheless 0.73 to 0.81 across models, so the relative ordering of burn extent was tracked well, and the deficit lies in metric anchoring.

Applying one calibration constant per model (k, the median ratio of reference to estimate) leaves the three models close together on this task (Figure 2d–f). The constants were 0.53 (0.49 to 0.60), 0.78 (0.72 to 0.86) and 0.85 (0.77 to 0.93) for Gemini 3.1 Pro, GPT-5.6 Sol and Fable 5, and the MAE of the averaged estimate fell from 17.82, 10.08 and 7.67 pp to 8.34 pp (7.00 to 9.62), 8.72 pp (7.41 to 10.11) and 7.34 pp (6.16 to 8.56). Errors that differed by more than a factor of two before rescaling lay within 1.4 pp of one another after it. Because k is estimated and evaluated on the same images, this figure is optimistic, so an LOOCV was run in which k was estimated on 152 images and applied to the held-out one, 153 times. The errors were then 8.42, 8.78 and 7.38 pp, so the convergence is not an artifact of in-sample fitting. A single multiplicative constant is also close to the best that a simple recalibration can do: allowing a free slope and an intercept instead removed only a further 1.2% to 2.8% of the original squared deviation.

Averaging repeated queries does not achieve the same thing, because it addresses the random component and not the systematic one. The MAE expected of a single query was 18.76, 12.30 and 7.91 pp, against 17.82, 10.08 and 7.67 pp for the mean of five queries: a gain of 0.95, 2.22 and 0.24 pp only. On the squared scale, on which the two components are additive, averaging the five runs reduced the squared error by 19.9%, 38.0% and 7.1%.

### 3.7. Random Error: Variability Across Repeated Identical Queries

Reproducibility across five identical queries differed markedly between models. For burned image area, ICC(A,1) was 0.67 (0.61 to 0.73) for Gemini 3.1 Pro, 0.57 (0.48 to 0.66) for GPT-5.6 Sol and 0.95 (0.93 to 0.96) for Fable 5, with mean within-image CVs of 29.6%, 35.9% and 13.4% (Table 1, Panel A). For TBSA the ICCs were 0.78 (0.73 to 0.83), 0.88 (0.85 to 0.91) and 0.95 (0.94 to 0.96), with CVs of 45.7%, 43.1% and 24.4% (Table 1, Panel B). For FST classification, Fleiss’ κ across runs was 0.70 (0.60 to 0.79), 0.60 (0.50 to 0.68) and 0.86 (0.79 to 0.92) (Table 2).

**Figure 3:**
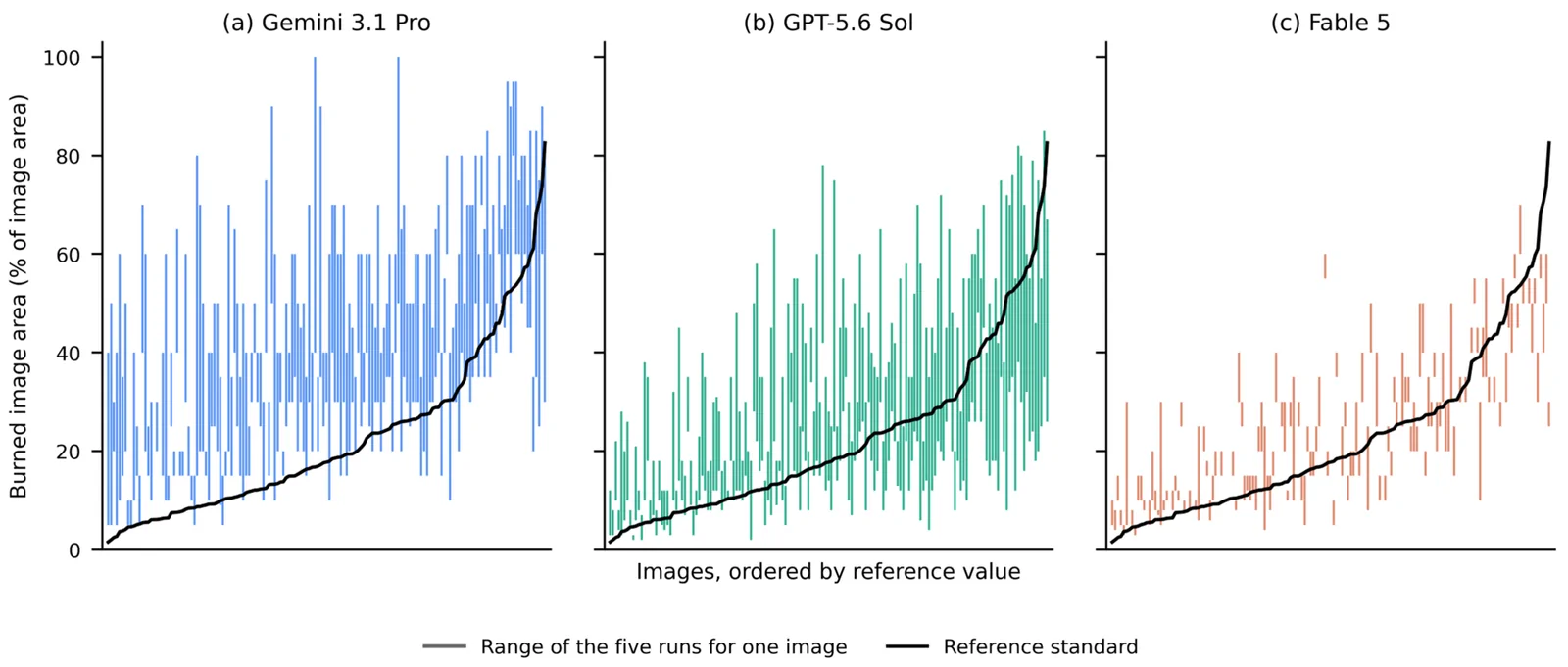
Variability across five identical queries, burned image area. Each vertical line represents one image and spans the minimum to the maximum of the five estimates returned by the same model for that image; a range of zero leaves no visible line. Images are ordered along the abscissa by increasing the reference value, shown as the solid black line. The median within-image range was 25.0 pp in (**a**) Gemini 3.1 Pro (interquartile range 15.0–35.0; maximum 65.0), 19.0 pp in (**b**) GPT-5.6 Sol (11.0–31.0; maximum 64.0) and 5.0 pp in (**c**) Fable 5 (3.0–10.0; maximum 25.0). pp = percentage points.

Expressed in the original unit, the same variability is more concrete (Figure 3). For burned image area the median within-image range (the largest minus the smallest of the five answers to an identical query) was 25.0 pp for Gemini 3.1 Pro (IQR 15.0–35.0; maximum 65.0), 19.0 pp for GPT-5.6 Sol (11.0–31.0; maximum 64.0) and 5.0 pp for Fable 5 (3.0–10.0; maximum 25.0). For one model the typical spread of repeated answers to the same photograph therefore exceeded the full 20-percentage-point width of the tolerance band within which an answer counts as correct. On the TBSA scale the corresponding medians were 2.6, 1.3 and 1.0 pp. The random error is not constant across the measurement range but grows with the value being estimated: for burned image area the within-image SD across the five runs correlated with the model’s own estimate at Spearman ρ = +0.44 for Gemini 3.1 Pro, +0.83 for GPT-5.6 Sol and +0.41 for Fable 5 (all *p* < 10^−6^). Aggregate performance moved with the runs as well: the SD of MAE across the five runs was 0.39 to 1.81 pp on Task 1, and the SD of Task 3 accuracy 2.5 to 4.0 pp.

### 3.8. Structure of the TBSA Agreement

**Figure 4:**
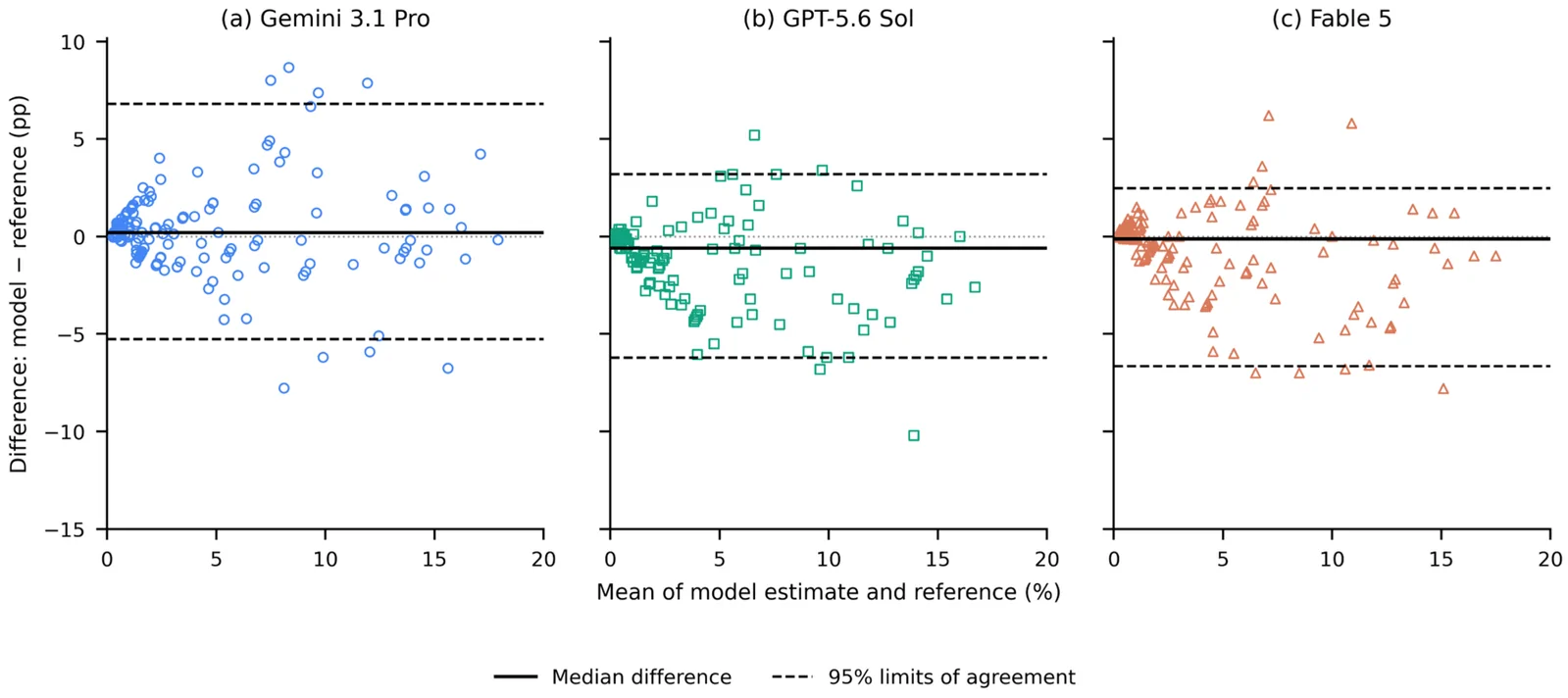
Bland–Altman analysis of the TBSA estimates for (**a**) Gemini 3.1 Pro, (**b**) GPT-5.6 Sol and (**c**) Fable 5 against the consensus physician reference. Each point represents one image; the difference is the mean of the five runs minus the reference, plotted against the mean of the two. The solid line is the median difference; the dashed lines are the non-parametric limits of agreement (the 2.5th and 97.5th percentiles of the differences). Median difference and limits of agreement were +0.20 pp (−5.26 to +6.80) in (**a**), −0.59 pp (−6.20 to +3.20) in (**b**) and −0.12 pp (−6.64 to +2.48) in (**c**). Positive values indicate an estimate above the physician reference. pp = percentage points; TBSA = total body surface area.

Bland–Altman analysis of the run-averaged TBSA estimates (Figure 4) gave median differences of +0.20 pp for Gemini 3.1 Pro, −0.59 pp for GPT-5.6 Sol and −0.12 pp for Fable 5, with limits of agreement of −5.26 to +6.80 pp, −6.20 to +3.20 pp and −6.64 to +2.48 pp. The differences departed from normality for every model (Shapiro–Wilk, all *p* < 0.001). The run-averaged estimate lay within roughly 6 to 7 pp of the physician reference in either direction.

### 3.9. FST Subgroups and the Burn-Extent Confounder

On the absolute error scale all three models performed less well on dark-skin images in both continuous tasks (Table 3, Panels A and B). For burned image area the MAE was 17.49 pp (15.84 to 19.27) against 24.19 pp (20.08 to 28.52) for Gemini 3.1 Pro, 11.53 pp (10.23 to 12.80) against 15.56 pp (11.94 to 19.58) for GPT-5.6 Sol and 7.03 pp (6.04 to 8.08) against 11.69 pp (7.85 to 16.06) for Fable 5. For TBSA the corresponding values were 1.83 against 2.72 pp, 1.61 against 2.72 pp and 1.37 against 2.36 pp. Within-tolerance rates were lower in the dark-skin subgroup for every model and both tasks, by 6.8 to 25.4 pp.

These differences reached significance for one of three models on burned image area and for all three on TBSA. On burned image area, Gemini 3.1 Pro showed a difference in median per-image error of −9.56 pp (95% CI −12.85 to −1.39; q = 0.006), while the other two reached q = 0.061. On TBSA the differences were −0.77 to −1.50 pp (q = 0.016 to 0.029). The dark-skin subgroup also contained larger burns: the median reference TBSA was 6.00% against 2.00% in the light-skin subgroup (Mann–Whitney *p* < 0.001), and on the image scale it was 20.18% against 16.82% (*p* = 0.106; Table 3, Panel D). The subgroup comparison of absolute error is therefore confounded by burn extent.

Adjusting the comparison for burn magnitude left none of these differences distinguishable from zero. With both reference values entered as covariates, the adjusted difference in mean absolute error between light and dark skin, on the mean rather than the median scale, was −4.37 pp (95% CI −9.30 to +0.70) for Gemini 3.1 Pro, −1.12 pp (−4.80 to +2.81) for GPT-5.6 Sol and −2.58 pp (−6.69 to +1.41) for Fable 5 on burned image area and +0.03 pp (−0.78 to +0.75), +0.00 pp (−0.53 to +0.58) and −0.13 pp (−0.77 to +0.54) on TBSA. The corresponding unadjusted differences in mean absolute error were −6.69, −4.02 and −4.66 pp and −0.89, −1.11 and −0.99 pp. With 29 dark-skin images the adjusted estimates are imprecise, so this is an absence of a demonstrable difference and not a demonstrated absence.

For FST classification, which does not depend on burn size, one difference was significant (Table 3, Panel C). Gemini 3.1 Pro classified 95.2% of light-skin images correctly against 72.4% of dark-skin images, a risk difference of 22.7 pp (95% CI 8.8 to 41.1; q = 0.003). The corresponding differences were +8.1 pp for Fable 5 (−1.8 to +25.2; q = 0.332) and −0.7 pp for GPT-5.6 Sol (−11.8 to +16.8; q = 1.000), the only model whose unadjusted point estimate did not favor light skin. Neither difference was significant. The same adjustment left this pattern unchanged: +23.4 pp (95% CI +6.3 to +40.6) for Gemini 3.1 Pro, +0.4 pp (−13.5 to +14.7) for GPT-5.6 Sol and +9.9 pp (−2.5 to +23.9) for Fable 5.

### 3.10. Model Comparisons

Differences between models were tested pairwise on the same 153 images (Table 4). On burned image area the omnibus comparison was significant (Friedman χ^2^ = 153.98, df = 2, *p* < 0.001), and all three pairwise comparisons survived correction (all q < 0.001), with median per-image paired differences in absolute error of +6.06 pp (95% CI +4.47 to +7.34) for Gemini 3.1 Pro versus GPT-5.6 Sol, +9.75 pp (+8.49 to +11.00) for Gemini 3.1 Pro versus Fable 5 and +3.17 pp (+2.11 to +4.49) for GPT-5.6 Sol versus Fable 5. On TBSA the omnibus comparison was also significant (Friedman χ^2^ = 10.13, df = 2, *p* = 0.006) but the differences were small: Fable 5 differed from both other models after correction, with median per-image paired differences in absolute error of +0.20 pp (+0.10 to +0.39) for Gemini 3.1 Pro versus Fable 5 and +0.10 pp (+0.03 to +0.30) for GPT-5.6 Sol versus Fable 5, both q = 0.001, whereas Gemini 3.1 Pro and GPT-5.6 Sol did not differ from each other at all, the median paired difference being +0.00 pp (−0.10 to +0.12; q = 0.431). On FST classification the omnibus test was significant (Cochran’s Q = 8.43, df = 2, *p* = 0.015) and one of three pairwise comparisons survived correction, with Fable 5 exceeding GPT-5.6 Sol by 7.2 pp (95% CI 2.0 to 12.3; q = 0.038).

Each model was also compared with the trivial classifier that always answers “light” (Table 4, Panel D). Gemini 3.1 Pro exceeded the baseline by 9.8 pp (95% CI +3.3 to +16.3; q = 0.009) and Fable 5 by 11.8 pp (+4.8 to +18.8; q = 0.006), whereas GPT-5.6 Sol exceeded it by 4.6 pp with an interval spanning zero (−3.8 to +12.9; q = 0.360). The observed accuracy of 85.6% for that model is thus not distinguishable from always predicting the majority class. Its discordant pairs were 25 dark-skin images classified correctly against 18 light-skin images misclassified.

## 4. Discussion

This benchmark evaluated three SOTA MLLMs on burn photographs under a repeated-inference design against one pixel-wise and two consensus physician reference standards. The results are best read as a decomposition of total measurement error into two components. Bias, the systematic component, shifts the distribution of a model’s answers away from the reference; noise, the random component, widens it without moving its center. On the squared scale the two are additive. Each has its own remedy: noise averages out over repeated queries, whereas bias does not and requires a correction factor estimated against reference data. A single accuracy figure reports their sum and identifies neither.

Several indicators are favorable when taken on their own: rank correlations of 0.73 to 0.81 between the run-averaged estimates and the pixel-wise reference indicate that the ordering of cases by burn extent is largely preserved. On TBSA the models sat 1.55 to 2.00 pp from the physician consensus on average, and FST classification reached 83.8% to 91.9%. Read alone, these figures would support a cautiously optimistic account.

### 4.1. Systematic Error: The Estimation Scale

Every model compressed the range, with slopes of 0.58 to 0.76 and positive intercepts of +10.0 to +22.2 pp, so that small burns were overestimated and large ones underestimated. That one multiplicative constant per model brings errors differing by more than a factor of two into a 1.4 pp range is the clearest expression of this: on this task the models differ far less in the rank information their outputs carry than in the scale on which they report a percentage, and the apparent ranking between them in accuracy is largely a calibration artifact. This is a statement about the reported numbers only: the design does not observe how a model delineates the burned region, and comparable errors after rescaling could arise from different internal representations.

In the prospective emergency-department study of a different model on real patients, regression of the model estimate on the expert-panel reference gave a slope of 0.71 with an R^2^ of 0.42, which the authors read as compression of the extremes toward the center of the distribution. The eighteen emergency physicians who rated the same images against the same reference showed no such compression, with a slope of 1.01 and an R^2^ of 0.88. Range compression is therefore unlikely to be an artifact of the present dataset, and the contrast with human raters on the same task argues that it reflects model behavior rather than an inherent property of the estimation task. In the present data the compression can also not be attributed to the reference: the reference is a fixed pixel count rather than a repeated measurement, so it contributes no replication noise, and the sub-unity slope is not regression dilution [20].

The same scoring behavior has been described outside burn assessment. In a benchmark of three frontier model families on an ordinal clinical rating scale, all three compressed their scores toward the middle of the range, over-predicting at the low end and under-predicting at the high end, an effect the authors term central tendency or endpoint compression. Two observations there bear on the present findings. In targeted ablations on one of the three models, few-shot exemplars spanning the full score range attenuated the compression without resolving it, and removing clinical terminology from the prompt did not reduce it at all, which argues against reading the compression observed here as an artifact of the single prompt configuration used in this study. And that same model assigned the top score more often to cases one level below the maximum than to cases at the maximum itself, indicating a scoring tendency rather than an inability to recognize extreme cases [43].

Why the scale should compress at all is not established. One proposal, advanced for ordinal clinical scoring rather than continuous estimation, is that alignment training internalizes the reluctance of human raters to assign extreme values. The persistence of the effect under score-balanced evaluation further argues against class imbalance as its sole explanation. The present data do not allow the underlying mechanism to be determined. The practical implication is one a leaderboard does not convey: a model whose scale is compressed but monotone is a candidate for post hoc calibration, whereas a model whose estimates bear no consistent relation to the reference is not. What such a correction can achieve is bounded: allowing a slope in addition to the single factor added almost nothing, so what remains after a full linear correction is not a further scale error but a residual that a linear map cannot reach [43].

### 4.2. Random Error and the Limits of Repetition

The same photograph does not yield the same answer, and repetition addresses only part of the problem. ICCs for burned image area ranged from 0.57 to 0.95 given identical prompts and images, and CVs reached 45.7% on the TBSA scale. In the original unit, the median spread between the largest and smallest of five answers to an identical query was 25.0 pp for one model, 19.0 pp for a second and 5.0 pp for the third, with individual images reaching 65 pp. For one model the typical spread exceeded the full 20 pp width of the tolerance band, which means that its reported accuracy reflects which answer it happened to give at least as much as its reading of the image. The intuitive remedy, querying several times and averaging, was tested on the same data and largely fails: it reduced the MAE by 0.24 to 2.22 pp only. Once the two components are separated this ceases to be surprising and becomes arithmetic. Averaging acts on the noise alone. The bias survives it unchanged, and here the bias is the larger of the two. On the squared scale, where the two are additive, averaging five answers removed between 7% and 38% of the total, so the greater part of it is a displacement that no number of repetitions can reach. Repetition and recalibration therefore do not substitute for one another. The random error is also not uniform across the measurement range but grows with the estimated value, so the interval within which a single answer may be expected to fall is widest exactly where the burn is largest. This also reinforces a concern that has been raised for single-run evaluations of stochastic systems: one query per case reports what the model answered on that occasion, not what it answers typically. Both studies [20,43] discussed in Section 4.1 queried each case once, a design that cannot detect this component at all [20,22,43].

### 4.3. FST Performance Against the Majority-Class Baseline

Because 81.0% of the images show a light skin tone, always answering “light” attains 81.0% accuracy. Tested against that classifier, two models clear it decisively, by 11.8 and 9.8 pp, while the third exceeds it by 4.6 pp with an interval spanning zero, gaining on dark skin about as much as it loses on light. Its accuracy of 85.6% is therefore largely class prevalence. That two models do clear the baseline shows the task is solvable from these photographs. All three accuracies exceed the baseline value, so the accuracy figures alone do not identify which models do so beyond chance. On the extent tasks, all three models erred more on dark-skin images, but the dark-skin subgroup also contained larger burns (a median reference TBSA of 6.00% against 2.00%), and absolute error scales with the magnitude of the estimated quantity, so that comparison is confounded by burn extent. After adjustment for both reference extents, none of the differences on the two extent tasks remained distinguishable from zero, though with 29 dark-skin images that is not evidence of equivalence. The one FST disparity the data do support is in classification itself, where one model fell from 95.2% correct on light skin to 72.4% on dark skin, a risk difference of 22.7 pp. This is consistent with the documented under-representation of darker skin tones in dermatological image resources and with the same limitation identified for burn-imaging datasets specifically. Limited skin-tone diversity was named as an open question by the prospective emergency-department evaluation, which could not address it. The present subgroup analysis is exploratory but speaks to it directly [16,20,27].

### 4.4. Interpretation of the Error Magnitudes

The direction of error on burned image area, systematic overestimation by all three models, parallels the well-documented tendency of clinicians at referring institutions to overestimate burn size [1,3]. The parallel extends beyond a shared direction: in one series of transferred patients the referring estimate exceeded the burn-center estimate by 4.3 pp on average for burns below 20% TBSA and fell short of it by 4.9 pp for burns at or above that threshold, which is the pattern the regression describes for the models here [44]. That pattern is not universal among human raters, however, as noted above [20]. The resemblance therefore holds against chart-based estimation in routine practice rather than against readers working from photographs under a standardized protocol. The parallel should not be pressed too far, because burned image area and TBSA are different quantities, and on TBSA two of the three models in fact underestimated relative to the physician reference. The two directions carry different risks. Underestimation leads, among other consequences, to insufficient fluid resuscitation, and because several national guidelines define referral criteria in part by TBSA thresholds, it can also delay transfer to a specialist center [45,46]. Overestimation leads to fluid volumes in excess of what the injury requires and to transfers and burn-center capacity taken up by patients who did not need them [1,4,20]. The observation that the direction of bias is not stable across tasks within one model is itself a caution against assuming that a single correction factor could serve across tasks.

To place the TBSA figures in a quantity burn clinicians work with directly, under the Parkland formula the resuscitation volume for the first 24 h is 4 mL × body weight in kilograms × %TBSA, so for an 80 kg adult each percentage point of TBSA error corresponds to 320 mL. The MAEs observed here, 1.55 to 2.00 pp, correspond arithmetically to roughly 500 to 640 mL in the calculated first-24-h volume for such a patient, and the limits of agreement for the run-averaged estimate, approximately −6 to +7 pp, to a span of some 4 L. These are conversions of an estimation error into the units of the formula, not observed prescribing errors: no fluid was prescribed and no patient was treated in this study. Because TBSA enters directly into every established resuscitation formula, whether Parkland, modified Parkland, Brooke, Cincinnati or Galveston, any estimation error translates proportionally into the calculated volume. At the limits of agreement the resulting difference is clinically relevant [4,47].

Taken together, within the limits of a single-image design without clinical context and the further limitations set out below, the results place current SOTA MLLMs some distance from the accuracy that would be required to inform downstream clinical judgment, let alone decisions on resuscitation volume or transfer. They were nevertheless the right systems to examine: GPAI models are at present the most widely used class of AI system and the largest single destination of private AI investment [48], so their behavior determines what clinicians and patients actually encounter, whether or not they were built for the task. In practice they are reached for outside clinical supervision as well, by clinicians informally and by laypeople photographing an injury and asking what to do. That is the setting in which the two error components matter most and are least detectable, since a user cannot tell an anchored answer from a displaced one, and it is where calibration and stability, among other things, would have to improve before such use could be considered informative.

Whether GPAI systems will eventually displace purpose-built tools for burn depth or burn extent assessment is open, and it touches a broader claim in machine learning, namely that general, data-driven and heavily scaled methods tend to overtake specialized, hand-engineered ones over time [49,50,51]. The present results do not yet support that claim for burn assessment. A further question would follow if they did: whether such systems remain usable on widely available hardware, which is what makes them low-threshold, or whether they come to require dedicated equipment in the way laser Doppler imaging does. Both questions can only be settled by repeated evaluation as the models change.

### 4.5. Limitations

The references for Tasks 2 and 3 were established in a joint consensus reading rather than by independent rating, so no separate rating series exist and neither inter-rater nor intra-rater reliability could be quantified. The Task 2 reference is an estimate compared with an estimate, obtained under a visible-field-only instruction, so the values are a whole-body TBSA extrapolated from one visible field rather than assessed over the whole patient. Its own precision is therefore unquantified, and small differences between models on this task should be read with that in mind. The task also compounds several judgments, since a whole-body percentage extrapolated from one visible field requires the burn to be delineated, the imaged region to be identified and its share of the body surface to be estimated, so an error on this task cannot be attributed to any one of them.

Because FST was assessed visually from single photographs, this analysis speaks to apparent pigmentation and to the equity of performance across visible skin tone, not to ultraviolet sensitivity or to Fitzpatrick phototype as originally defined. Visual assignment on this scale is itself subjective and inconsistently documented [37].

The dark-skin-tone subgroup comprises 29 images. That analysis is exploratory and was not powered for equivalence, and non-significant results are not evidence of no difference (smallest non-significant q = 0.061). No relative error scale is reported: it would be one candidate for removing the burn-size confounding from the FST comparison, but it is unstable precisely there, since 43 of the 124 light-skin images have a TBSA reference below 1% and the smallest is 0.10%, where a 0.4 pp deviation becomes a 400% relative error. Regression adjustment on the absolute scale was used instead, which leaves the comparison without a demonstrable difference rather than with a resolved one.

All findings come from a single image set and have not been validated externally. The calibration constant in particular was estimated and evaluated on the same images, though the LOOCV indicates that the resulting optimism is small. It addresses in-sample optimism only and does not establish that the constant would transfer to other image sets, acquisition conditions or later model versions. The presence of the images in the pretraining corpora of the evaluated models cannot be excluded, and any resulting advantage would favor the models. They were acquired under heterogeneous illumination, cameras and camera-to-subject distances, none of which could be controlled or analyzed as a covariate. The evaluation uses one photograph per case, without clinical context (age, mechanism, or time since injury) and without the multiple views or tactile examination available at the bedside. Multiple views or three-dimensional surface capture would additionally address the projection error that arises when a curved surface is assessed from a single two-dimensional view. Burn depth, the second quantitative variable governing early management, was not assessed, and all three tasks were elicited within one prompt per image, so coupling between the answers cannot be excluded and the three results should not be read as independent assessments of the same image.

Because neither CLAIM nor STARD-AI applies in full, several item groups have no counterpart in a study of this kind, neither in its design nor in the material it examines: participant recruitment and setting, a clinical reference standard, the interval between the index test and reference standard, a participant flow diagram, indeterminate results, and a defined intended use. No sample size calculation was performed. Finally, three models at one point in time are a narrow sample of a fast-moving field. Each was queried in a single prompt configuration through its consumer web interface, where sampling behavior is set by the vendor and the served version may change without notice. That interface was chosen deliberately, since it is how these systems are actually used [28,29].

## 5. Conclusions

Across 153 burn photographs and five independent runs, three SOTA MLLMs tracked the relative extent of a burn reasonably well, but neither anchored it on an absolute scale nor answered consistently when the same question was put repeatedly. The failure of scale was systematic enough that one multiplicative constant per model largely removed the accuracy differences between the models on burned image area. Read as measurement error, the two shortfalls are distinct: bias displaces the distribution of answers and is in principle correctable against reference data, whereas noise widens it and can only be averaged down, at a cost in queries and with a modest return. A single accuracy figure combines the two without separating them, and one obtained from a single query per case cannot establish what a model typically answers at all. Performance across skin tones was uneven and model-specific, and where it appeared unequal on the extent tasks it did not survive adjustment for burn size. On burn extent, and within the limits of the present study design, current SOTA MLLMs do not reach a level of performance that would qualify them for consideration in clinical use. Whether they will is in part a question of how their calibration and their stability develop, and neither is visible in a single answer. External validation, acquisition conditions, the absence of clinical context, projection error from a single or non-standardized view, and the burn depth that would have to be assessed alongside extent all pose further challenges.

## Figures and Tables

**Table 1:** Continuous estimation tasks.

Model	MAE, pp	Signed Error, pp	Within Tol., %	Error Spread, pp	ICC(A,1)	CV, %	Range, pp
Panel A. Task 1: burned image area; tolerance ± 10 pp							
Gemini 3.1 Pro	18.76 (17.10–20.45)	+16.80 (14.84–18.86)	32.5 (27.8–37.6)	−12.5 to +54.2	0.67 (0.61–0.73)	29.6 (26.8–32.7)	25.0 (15.0–35.0); 65.0
GPT-5.6 Sol	12.30 (11.06–13.57)	+4.49 (2.52–6.44)	54.5 (49.4–59.9)	−33.8 to +37.8	0.57 (0.48–0.66)	35.9 (33.7–38.3)	19.0 (11.0–31.0); 64.0
Fable 5	7.91 (6.84–9.13)	+2.20 (0.60–3.77)	70.2 (63.8–76.1)	−20.5 to +23.2	0.95 (0.93–0.96)	13.4 (11.3–15.8)	5.0 (3.0–10.0); 25.0
Panel B. Task 2: TBSA; tolerance ± 2 pp							
Gemini 3.1 Pro	2.00 (1.70–2.32)	+0.29 (−0.09 to 0.68)	71.6 (66.4–76.6)	−7.0 to +10.0	0.78 (0.73–0.83)	45.7 (41.4–50.2)	2.6 (1.1–6.5); 17.5
GPT-5.6 Sol	1.82 (1.54–2.13)	−1.11 (−1.47 to −0.79)	69.5 (63.3–75.4)	−7.0 to +4.0	0.88 (0.85–0.91)	43.1 (39.6–46.5)	1.3 (0.5–3.0); 13.0
Fable 5	1.55 (1.29–1.84)	−0.72 (−1.07 to −0.39)	77.5 (71.1–83.1)	−7.0 to +3.0	0.95 (0.94–0.96)	24.4 (20.6–28.4)	1.0 (0.5–1.5); 13.5

MAE = mean absolute error; CV = coefficient of variation; ICC = intraclass correlation coefficient; pp = percentage points; TBSA = total body surface area. *n* = 153 images per model and panel. Values are mean (95% CI) unless stated. Positive signed errors indicate estimates above the reference. Error spread: 2.5th–97.5th percentile of the 765 single-query signed errors per model. Range: median (IQR); maximum of the within-image maximum minus minimum across the 5 runs; the parentheses in this column give the IQR, not a CI. ICC(A,1) and CV describe variation across the 5 runs of the same image, not agreement with the reference; MAE, signed error and within-tolerance rate are computed within each run and then averaged. Panels are not comparable: the two quantities differ in denominator and in tolerance. CIs are case-level bootstrap intervals, except the ICC [38].

**Table 2:** FST classification performance.

Model	Accuracy, %	MV acc., %	Sens., %	Spec., %	PPV, %	NPV, %	F1, %	Fleiss’ κ
Gemini 3.1 Pro	90.2 (86.4–93.6)	90.8	73.8 (63.0–84.2)	94.0 (90.4–97.1)	74.0 (59.2–87.2)	93.9 (90.4–97.0)	73.7 (62.7–82.8)	0.70 (0.60–0.79)
GPT-5.6 Sol	83.8 (79.6–87.8)	85.6	82.1 (70.7–92.0)	84.2 (79.5–88.7)	55.7 (42.1–67.0)	95.3 (91.6–98.2)	66.0 (53.4–75.2)	0.60 (0.50–0.68)
Fable 5	91.9 (88.1–95.3)	92.8	84.1 (71.6–94.8)	93.7 (89.8–97.0)	76.5 (62.9–88.1)	96.2 (92.9–99.0)	79.9 (69.1–88.2)	0.86 (0.79–0.92)
Majority-class baseline	81.0	81.0	0.0	100.0	—	81.0	0.0	—

PPV = positive predictive value; NPV = negative predictive value; MV = majority vote; *n* = 153 images per model. Values are mean (95% CI) unless stated. Positive class = dark skin tone (FST IV–VI), *n* = 29; negative class = light, *n* = 124. Baseline = always predicting the light skin tone; tested against each model in Section 3.10. MV accuracy and Fleiss’ κ are computed across the 5 runs; all other values are run-averaged. Balanced accuracy and the Matthews correlation coefficient are reported in Section 3.5.

**Table 3:** FST subgroup performance and the burn-extent confounder.

Model	Light (*n* = 124)	Dark (*n* = 29)	*p*	q	Effect Size (95% CI)
Panel A. Task 1: burned image area; mean absolute error per image, pp					
Gemini 3.1 Pro	17.49 (15.84–19.27)	24.19 (20.08–28.52)	0.002	0.006	−9.56 (−12.85 to −1.39)
GPT-5.6 Sol	11.53 (10.23–12.80)	15.56 (11.94–19.58)	0.061	0.061	−3.41 (−7.76 to +1.40)
Fable 5	7.03 (6.04–8.08)	11.69 (7.85–16.06)	0.044	0.061	−1.51 (−8.78 to +1.33)
Panel B. Task 2: TBSA; mean absolute error per image, pp					
Gemini 3.1 Pro	1.83 (1.53–2.16)	2.72 (1.94–3.51)	0.029	0.029	−0.77 (−2.44 to +0.05)
GPT-5.6 Sol	1.61 (1.34–1.90)	2.72 (1.98–3.54)	0.007	0.016	−1.50 (−2.39 to −0.23)
Fable 5	1.37 (1.11–1.65)	2.36 (1.64–3.16)	0.011	0.016	−0.80 (−2.40 to +0.00)
Panel C. Task 3: FST classification; majority-vote classification correct, % (*n*/*N*). Effect size = risk difference					
Gemini 3.1 Pro	95.2 (118/124)	72.4 (21/29)	<0.001	0.003	+22.7 pp (+8.8 to +41.1)
GPT-5.6 Sol	85.5 (106/124)	86.2 (25/29)	1.000	1.000	−0.7 pp (−11.8 to +16.8)
Fable 5	94.4 (117/124)	86.2 (25/29)	0.222	0.332	+8.1 pp (−1.8 to +25.2)
Panel D. Reference burn extent by subgroup; median %					
Burned image area	16.82	20.18	0.106	—	—
TBSA	2.00	6.00	<0.001	—	—

CI = confidence interval; FST = Fitzpatrick skin tone; TBSA = total body surface area; pp = percentage points. q = *p* value after Benjamini–Hochberg correction for false discovery rate. Subgroups were defined by the physician reference. Panels A, B and D: Mann–Whitney U; Panel C: Fisher’s exact test. CIs in Panels A and B are bootstrap intervals for the difference of the medians, so an interval may include zero where the test reaches significance. In Panels A and B the subgroup columns are mean absolute errors, and the effect size is the difference of the median per-image errors; the two are therefore not arithmetically related. Negative values indicate a larger error in the dark-skin subgroup; in Panel C, positive values indicate higher accuracy in the light-skin subgroup. Panels A and B are confounded by burn extent (Panel D). Estimates adjusted for burn magnitude are reported in the text of this section.

**Table 4:** Statistical comparisons.

Comparison	Test	*p*	q	Effect Size (95% CI)
Panel A. Task 1: burned image area; paired on the same 153 images, per-image absolute error				
All three models	Friedman, χ^2^ = 153.98, df = 2	<0.001	—	—
Gemini 3.1 Pro vs. GPT-5.6 Sol	Wilcoxon	<0.001	<0.001	+6.06 pp (+4.47 to +7.34)
Gemini 3.1 Pro vs. Fable 5	Wilcoxon	<0.001	<0.001	+9.75 pp (+8.49 to +11.00)
GPT-5.6 Sol vs. Fable 5	Wilcoxon	<0.001	<0.001	+3.17 pp (+2.11 to +4.49)
Panel B. Task 2: TBSA; paired on the same 153 images, per-image absolute error				
All three models	Friedman, χ^2^ = 10.13, df = 2	0.006	—	—
Gemini 3.1 Pro vs. GPT-5.6 Sol	Wilcoxon	0.431	0.431	+0.00 pp (−0.10 to +0.12)
Gemini 3.1 Pro vs. Fable 5	Wilcoxon	<0.001	0.001	+0.20 pp (+0.10 to +0.39)
GPT-5.6 Sol vs. Fable 5	Wilcoxon	<0.001	0.001	+0.10 pp (+0.03 to +0.30)
Panel C. Task 3: FST classification, model against model; per-image majority vote				
All three models	Cochran’s Q, Q = 8.43, df = 2	0.015	—	—
Gemini 3.1 Pro vs. GPT-5.6 Sol	McNemar	0.115	0.173	+5.2 pp (−0.4 to +10.9)
Gemini 3.1 Pro vs. Fable 5	McNemar	0.508	0.508	−2.0 pp (−5.8 to +1.9)
GPT-5.6 Sol vs. Fable 5	McNemar	0.013	0.038	−7.2 pp (−12.3 to −2.0)
Panel D. Task 3: each model against the majority-class baseline; per-image majority vote				
Gemini 3.1 Pro vs. baseline	McNemar	0.006	0.009	+9.8 pp (+3.3 to +16.3)
GPT-5.6 Sol vs. baseline	McNemar	0.360	0.360	+4.6 pp (−3.8 to +12.9)
Fable 5 vs. baseline	McNemar	0.002	0.006	+11.8 pp (+4.8 to +18.8)

CI = confidence interval; FST = Fitzpatrick skin tone; TBSA = total body surface area; pp = percentage points. q = *p* value after Benjamini–Hochberg correction for false discovery rate. All comparisons are paired on the same 153 images. Omnibus tests carry no effect size. Effect sizes in Panels A and B are the median per-image paired difference in absolute error; positive values indicate a larger error in the first-named model. In Panels C and D they are risk differences in the proportion classified correctly; positive values indicate better classification by the first-named model. Panel D: baseline = always predicting the light skin tone. Benjamini–Hochberg was applied within each panel; omnibus tests were not corrected. Pooling Panels C and D would give q = 0.018, 0.432 and 0.013 in Panel D and q = 0.025 for GPT-5.6 Sol vs. Fable 5 in Panel C.

## Data Availability

The dataset analyzed in this study is publicly available; see [30] for repository details.

## Supplementary Materials

The following supporting information can be downloaded at https://www.mdpi.com/article/10.3390/bioengineering13091000/s1, File S1: Prompt used for all model queries.

## Abbreviations

The following abbreviations are used in this manuscript:

API	application programming interface
CI	confidence interval
CNN	convolutional neural network
CV	coefficient of variation
FST	Fitzpatrick skin tone
GPAI	general-purpose artificial intelligence
ICC	intraclass correlation coefficient
IQR	interquartile range
LOOCV	leave-one-out cross-validation
MAE	mean absolute error
MCC	Matthews correlation coefficient
MLLM	multimodal large language model
MV	majority vote
NPV	negative predictive value
pp	percentage points
PPV	positive predictive value
SD	standard deviation
SOTA	state of the art
TBSA	total body surface area
