# Supplementary material for: Burn Extent and Fitzpatrick Skin Tone Assessment from Clinical Photographs: Systematic and Random Error in Multimodal Large Language Models
Source: Bioengineering (Basel). 2026 Aug 28;13(9):1000. doi: 10.3390/bioengineering13091000 (PMC13603288; doi:10.3390/bioengineering13091000)
Supplement: Supplementary file 1 [file bioengineering-13-01000-s001.zip › bioengineering-4511068-supplementary.pdf]

The photograph shows a burn injury.

Report three values, in this order:

1. Burned image area: the percentage of the TOTAL image area (the whole photograph, including background) covered by burned skin. Count the burned region only.

2. TBSA: the percentage of the WHOLE-BODY surface area represented by the burn visible in this photograph. Map the visible burn onto the whole body. Assume there are no burns outside the visible field.

3. Skin type: light (Fitzpatrick I-III) = l or dark (Fitzpatrick IV-VI) = d

RULES:

- Exactly three lines, one value per line, in the order above.

- Items 1 and 2: a single number, decimals allowed, no % sign, no range.

- Item 3: the single letter l or d.

- Give a best estimate for every item; leave nothing blank.

- No explanations, no labels, no extra text.
